# Supplementary material for: Ultrafast magnetic dynamics in insulating YBa2Cu3O6.1 revealed by time resolved two-magnon Raman scattering
Source: Nat Commun. 2020 May 21;11:2548. doi: 10.1038/s41467-020-16275-9 (PMC7242324; doi:10.1038/s41467-020-16275-9)
Supplement: Supplementary file 1 — Supplementary Information [file 41467_2020_16275_MOESM1_ESM.pdf]

Supplementary Information for Ultrafast magnetic dynamics in insulating $YBa_2Cu_3O_{6.1}$ revealed by time resolved two-magnon Raman Scattering

Yang *et al*

Supplementary Note 1: Effect of self-pumping

Self-pumping is a well-known effect in time-resolved Raman scattering whereby photons from the probe laser pulse pump the system before the Raman scattering occurs [1, 2]. This process is quadratic as a function of laser power, whereas Raman scattering by itself is linear in laser power. As a result, making measurements with the probe alone (without the pump) at different laser powers will result is spectra that look more and more pumped as the power increases. Self-pumping probes timescales of less than the probe pulse duration, which in our case is about 40 fs. Supplementary Figure 1 compares Raman scattering cross section measured with the pump laser alone at a small laser power and the larger laser power used in most time-resolved measurements discussed above. A small reduction of the two-magnon peak intensity and a small increase of the low-energy part of the spectrum at the higher laser power originates from self-pumping. This effect is much smaller than the effect of the IR pump.

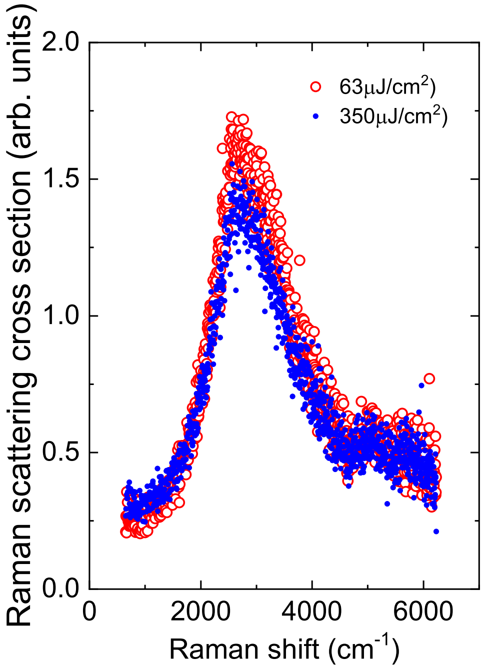

Supplementary Figure 1: Effect of self-pumping. Blue dots represent the two-magnon Raman spectrum obtained with the same pump fluence as the data in Figures 2-5 of the main text. Red circles represent the data obtained with a smaller fluence. Scattering intensities were divided by laser power and counting time.

Supplementary Note 2: Two-magnon fitting procedure

In order to extract the center position and width of the 2M peak, we fitted to a phenomenological model. First, we estimated the background at -1000 fs by fitting the points below 1000 cm^{-1} and above 5000 cm^{-1} to a cubic polynomial. Supplementary Figure 2 illustrates this process. For fits at all other delay times with the same fluence, we fit the points above 5000 cm^{-1} to a constant amplitude times the cubic polynomial found at -1000 fs. Fixing the shape of the background and only allowing it to vary in amplitude over time significantly reduced the uncertainty in the fitted peak positions and widths.

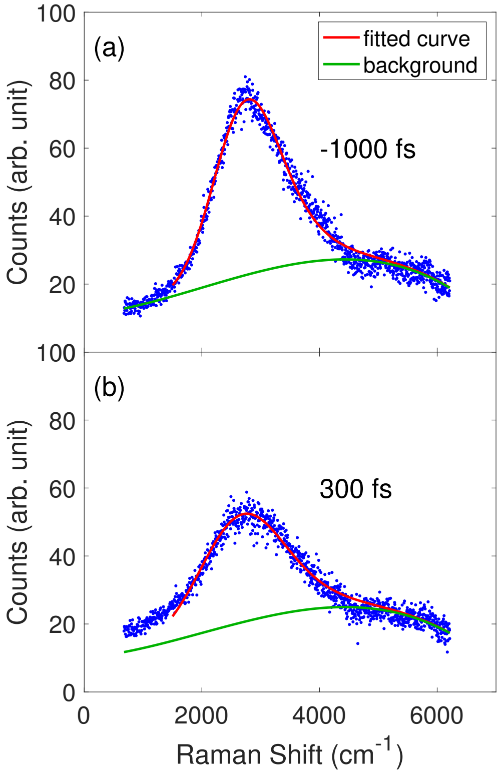

Supplementary Figure 2: Raw data with fitted curves and backgrounds at -1000 fs (a) and 300 fs (b). At -1000 fs, the background is a cubic polynomial fitted to the points below 1000 cm^{-1} and above 5000 cm^{-1}. At all other delay times (including at 300 fs shown here), the background was the same cubic polynomial, allowed to vary only by an overall amplitude to fit the points above 5000 cm^{-1}. At all delay times, points below 1500 cm^{-1} were excluded from fitting to the exponentially-modified Gaussian.

The remaining curve after background subtraction was fitted to an exponentially-modified Gaussian in order to account for the asymmetry of the peak. When performing the fits, we excluded points below 1500 cm^{-1} because the increase in spectral weight at positive delay times was not fitted well by our chosen lineshape and we were more interested in the position and width of the main 2M peak. The exponentially-modified Gaussian is a normal Gaussian convolved with a single exponential:

$$f(x) = A \times \exp\left(\frac{\lambda}{2}\left(2\mu + \lambda\sigma^2 - 2x\right)\right) \operatorname{erfc}\left(\frac{\mu + \lambda\sigma^2 - x}{\sqrt{2}\sigma}\right). \quad (1)$$

Here, $\operatorname{erfc}(z)$ is the complementary error function, A is an overall amplitude, λ is the decay constant for the underlying exponential, and μ and σ are the mean and standard deviation of the underlying Gaussian, respectively. In order to reduce the number of free parameters, we fit the spectrum at -1000 fs with all four parameters free and then used that fitted value of λ for all other fits. So the three free parameters for all fits were A, μ, and σ.

Note that the true peak position and width are not parameters of this equation, but depend on multiple parameters. For the peak position, we used an exact formula for the point at which the derivative of the curve vanishes:

$$x_{\text{max}} = \mu + \lambda\sigma^2 - \operatorname{sgn}(\lambda)\sqrt{2}\sigma \times \operatorname{erfcxinv}\left(\frac{1}{|\lambda|\,\sigma}\sqrt{\frac{2}{\pi}}\right), \quad (2)$$

where $\operatorname{erfcxinv}(z)$ is the inverse of the scaled complimentary error function. We calculated the approximate full-width half-maximum (FWHM) in analogy with the FWHM for a normal Gaussian:

$$\text{FWHM} \approx 2\sqrt{2\log 2}\left(\sigma^2 + \frac{1}{\lambda^2}\right)^{\frac{1}{2}}, \quad (3)$$

where the expression in parentheses is the variance of the exponentially modified Gaussian distribution.

Skewness is a statistical measure of the asymmetry of a distribution. It is dimensionless and a perfectly symmetric peak has a skewness of zero. For the exponentially-modified Gaussian distribution, the skewness is given by

$$\text{Skewness} = \frac{2}{\sigma^3\lambda^3}\left(1 + \frac{1}{\sigma^2\lambda^2}\right)^{-3/2}. \quad (4)$$

The error bars in Figure 5 of the main text for the peak center and width are the calculated 95% confidence intervals for μ and $2\sqrt{2\log 2}\sigma$, respectively. Using $\delta\mu$ for the error bar on the peak center is an approximation, which neglects the complicated dependence of x_{max} on σ.

Supplementary References

[1] H. Yan, D. Song, K. F. Mak, I. Chatzakis, J. Maultzsch, and T. F. Heinz, Time-resolved Raman spectroscopy of optical phonons in graphite: Phonon anharmonic coupling and anomalous stiffening, *Phys. Rev. B* **80**, 121403 (2009).

[2] J.A. Yang, S. Parham, D. Dessau, and D. Reznik, Novel electron-phonon relaxation pathway in graphite revealed by time-resolved Raman Scattering and Angle-Resolved Photoemission Spectroscopy, *Scientific Reports* **7**, 40876 (2017).
